# Annotations

**Published:** 1896-08-15

**Authors:** 


### Annotations.

### Local Authorities and Food Adulteration.

Mr. Arthur Giles, Secretary to the Federation of Grocers' Associations, writes to the effect that an article which recently appeared in The Hospital on "Margarine: Science and Selfishness," hardly does justice to the particular trades which he represents, that is, the grocers and associated trades. It was stated in our pages, on the authority of a Select Committee's report, that the local authorities of some of our towns and districts being largely composed of tradesmen, there was often difficulty in getting the Adulteration Acts carried out; and it was insisted that if such were the case, there was a grave reflection upon the honesty and straightforwardness of tradesmen as a class. Mr. Giles says we have been "misled." He insists, on the contrary, that "honest tradesmen" are very anxious to have the Adulteration Acts enforced, which we can well believe; and that, moreover, the Metropolitan Grocers' Association has itself undertaken no fewer than fifteen prosecutions under the Acts, which prosecutions the local authorities had refused to carry out. All this is, no doubt, so. But Mr. Giles only touches the fringe of the subject. Nobody supposes that "honest tradesmen" encourage adulteration, or that "associations of tradesmen" do so in their corporate capacity. What the public desires to know is this, whether the proportion of tradesmen who knowingly or carelessly deal in adulterated articles is large or small; and whether, where the tradesman element is strong in local authorities, the Adulteration Acts are, as a matter of fact, more or less of a dead letter. On these points, as we have said, Mr. Giles does not help us to any light. His two contentions, that honest tradesmen will act honestly, and that grocers' associations, as such, do not encourage knavery, we cordially endorse. If he will address himself in detail to the question whether or not in actual fact the tradesman element on the whole throughout the United Kingdom offers real resistance to the working of the Adulteration Acts, we shall be glad to hear from him again.

### About Vinegars.

There is nothing the scientific medical man likes better than to know "all and everything" about the food and the physic which he is daily ordering for his patients. Vinegar is one of the commonest of all our table condiments, and one of the most uncertain as to its quality. A new vinegar is claiming the suffrages of the public, and it is made from dates. Now, we have no intention whatever of giving a certificate of merit to any particular variety of date vinegar; but that dates, being highly sugary fruits and of fine flavour, should theoretically constitute the basis of excellent vinegar is obvious. Vinegars there be which are made from malt; others which are made from grapes; and others again—and their name is legion—which are made from—we know not what. In converting dates into vinegar the first thing to be done is to crush and mix them with water, and thus to form a pulp. The compression of the pulp is the next step; and then follows the sterilisation of the fluid of compression. After this, a specially-prepared yeast is added to the liquid in fermenting vats, and "date wine" is produced. Date vinegar is, therefore, a "wine" vinegar, not a vinegar of malt, or some other substance undefined. The last stage is the acetification of the wine. This, as everybody knows, is effected by an oxidation of the alcohol through the agency of the fungus *Mycoderma aceti.* The effect of the action of the mycoderma is to turn the wine "sour," or, in other words, to convert it into vinegar. Many people are under the impression that depth of colour is a mark of strength in vinegar. That is not so. Quite pure acetic acid is colourless: and the finest vinegars are light in colour and of a beautiful clearness and transparency. Date vinegar is very light in colour, and almost twice the strength of many of the brown vinegars. When properly prepared, it has a somewhat rare combination of merits: it is pure, strong, of fine aroma and flavour, and, last, but not least, is quite cheap. All this should commend date vinegar to general acceptance.

### Infantile Indigestion.

The dangers attending the artificial rearing of infants are well recognised, and a very large literature has grown up on the subject. It is curious, however, to notice how little attention has been paid to the indigestion which so often occurs in breast-fed infants, and is, in fact, in a large number of cases, the reason which leads mothers to give up suckling and revert to bottle-feeding, on the plea that her milk "disagrees." Probably a tenth part of the trouble involved in looking after bottles and in sterilising and otherwise preparing milk, if expended in rectifying the ailments which cause a mother's milk to disagree, would enable her to perform her natural functions, and to become a mother in a much wider sense than when she limits her function, as is so frequently the case, to the mere bearing of children. In a paper lately read before the Edinburgh Obstetrical Society by Dr. James Carmicheal, he points out that the indigestion of mother's milk is more frequently intestinal than gastric, diarrhœa being more common than vomiting, this being largely due to indigestion of the fatty and proteid elements of the milk. On inquiry as to the causes of this, we find, first, that it may arise from insanitary conditions, which may affect both the mother and the child, such as low-lying, damp dwelling-houses, effluvia from drains, or malaria poison. Very often, however, the defect is purely maternal. Debility is a frequent cause. Too frequent pregnancies, for example, may render a mother quite unable to produce healthy and nourishing milk for her later children, although she may have suckled her first child perfectly well. Inefficient food among poor women, and overwrought nervous system among the over-educated, may produce the same inability. Then ill health of any kind in the mother is apt to prove a hindrance to suckling, and if this ill health be of a nature that can be removed the function may be restored. Irregular suckling is, however, one of the commonest causes of indigestion in babies. This often arises from over anxiety about the child, and from the bad habit of giving it the breast whenever it cries. Children, like grown up people, always thrive best when fed with strict regularity.

